# Systematic Comparison of Computational Tools for Sanger Sequencing-Based Genome Editing Analysis

**DOI:** 10.3390/cells13030261

**Published:** 2024-01-30

**Authors:** Kanae Aoki, Mai Yamasaki, Riku Umezono, Takanori Hamamoto, Yusuke Kamachi

**Affiliations:** School of Engineering Science, Kochi University of Technology, Kami 782-8502, Japan275086d@gs.kochi-tech.ac.jp (M.Y.);

**Keywords:** ICE, CRISPR–Cas, DECODR, indel analysis, SeqScreener, TIDE

## Abstract

Successful genome editing depends on the cleavage efficiency of programmable nucleases (PNs) such as the CRISPR–Cas system. Various methods have been developed to assess the efficiency of PNs, most of which estimate the occurrence of indels caused by PN-induced double-strand breaks. In these methods, PN genomic target sites are amplified through PCR, and the resulting PCR products are subsequently analyzed using Sanger sequencing, high-throughput sequencing, or mismatch detection assays. Among these methods, Sanger sequencing of PCR products followed by indel analysis using online web tools has gained popularity due to its user-friendly nature. This approach estimates indel frequencies by computationally analyzing sequencing trace data. However, the accuracy of these computational tools remains uncertain. In this study, we compared the performance of four web tools, TIDE, ICE, DECODR, and SeqScreener, using artificial sequencing templates with predetermined indels. Our results demonstrated that these tools were able to estimate indel frequency with acceptable accuracy when the indels were simple and contained only a few base changes. However, the estimated values became more variable among the tools when the sequencing templates contained more complex indels or knock-in sequences. Moreover, although these tools effectively estimated the net indel sizes, their capability to deconvolute indel sequences exhibited variability with certain limitations. These findings underscore the importance of judiciously selecting and using an appropriate tool with caution, depending on the type of genome editing being performed.

## 1. Introduction

Genome editing using CRISPR–Cas systems (clustered regularly interspaced short palindromic repeats and CRISPR-associated proteins) has revolutionized modern biological research due to its high efficiency and ease of use compared to other programmable nucleases (PNs) such as ZFNs and TALENs [1,2]. The two main components of the CRISPR–Cas system are the Cas proteins, which are responsible for cleaving DNA, and CRISPR RNAs (crRNAs, also called guide RNAs or gRNAs), which direct catalytic activity against target DNA through complementarity between the spacer segment of the crRNA and target DNA. CRISPR–Cas systems are highly programmable because they allow for the replacement of spacer sequences of crRNAs. However, the cleavage efficiency of the CRISPR–Cas ribonucleoprotein (RNP) complex can vary among gRNAs [3,4]. Therefore, successful genome editing relies on the careful selection of gRNAs, and it is crucial to pre-evaluate the cleavage efficiency of candidate gRNAs for efficient genome editing. This is typically achieved by measuring the degree of insertions and deletions (indels) induced by the CRISPR–Cas complex at the target sites [5].

Several strategies have been developed to analyze indel frequencies; however, each strategy has specific limitations [5,6,7]. Mismatch cleavage assays, high-resolution melting analysis (HRMA), and heteroduplex mobility assays are based on the cleavage or modified migration of heteroduplexes formed by mutated and wild-type (wt) DNA strands [8,9,10]. These methods have been widely used for the preliminary screening of gRNA activity due to their simplicity and low cost. The most popular among these techniques utilizes T7 endonuclease 1 (T7E1) or Surveyor nuclease to cleave mismatches formed between modified and unmodified DNA strands [11,12]. Despite these advantages, mismatch cleavage assay signals have been shown to be more strongly associated with indel complexity than with indel frequency, resulting in underestimation, especially for samples with a single dominant indel [6]. Thus, indel detection methods that depend on sequencing technologies have recently been developed. Among them, Sanger sequencing of subcloned DNAs derived from PCR amplicons was initially used [13,14]. High-throughput sequencing of PCR amplicons has also been employed successfully [15,16]; however, this approach is expensive and requires more time than other approaches. Additionally, a method called Indel Detection by Amplicon Analysis (IDAA) has been developed to detect indels, in which labeled PCR amplicons are subjected to capillary electrophoresis for fragment analysis [14], although nucleotide sequence change information could not be obtained.

Concurrently, a computational tool called Tracking the Indels by Decomposition (TIDE) was developed. TIDE analyzes Sanger sequencing trace data of PCR amplicons to estimate the frequency of editing efficiency and indel distribution [17]. Subsequently, similar computational tools have been developed including ICE (Inference of CRISPR Edits) [18], DECODR (Deconvolution of Complex DNA Repair) [19], and the SeqScreener Gene Edit Confirmation App (an online tool provided by Thermo Fisher Scientific). These computational tools rely on decomposition algorithms to identify the indel spectrum such as the number of indels, indel size, and total indel frequency. They achieve this by comparing Sanger sequencing trace data from the wild-type control and genome-edited sample sequences. While the algorithms share many features, each tool has specific modifications that may result in different outputs.

These computational tools have been shown to outperform mismatch cleavage assays in terms of accuracy and quantitative capability [6] and have been successfully used in numerous studies [5]. Although these tools have been shown to provide indel profiles similar to those obtained by high-throughput sequencing [6,18,19], the accuracy of indel frequency estimation has not been extensively investigated. Furthermore, a recent report indicated that TIDE, ICE, and DECODR produced widely divergent indel frequency data from the same samples derived from CRISPR–Cas9-induced indels in mouse tumor models [20]. Therefore, in this study, we quantitatively compared the performances of these four computational tools using artificial sequencing templates with defined indels. For this purpose, we first cloned various indels that had been induced by CRISPR–Cas9 or CRISPR–Cas12a at several zebrafish gene loci. By using Sanger sequencing trace data obtained from various combinations of predetermined indels, we quantitatively assessed the performance of these computational tools. We demonstrated that these tools were able to estimate indel frequencies with reasonable accuracy when indels had a few base changes and indel frequencies were in the midrange. However, variations in estimated values became more pronounced among the tools when the samples contained more complicated indels or when the indel frequencies were in a low or high range. Among the four tools, DECODR provided the most accurate estimations of indel frequencies for the majority of samples, which was consistent with the findings of a recent report [20]. While all four tools accurately estimated the net indel sizes, DECODR was found to be the most useful when one wishes to identify indel sequences. We also assessed the performance of these computational tools for estimating the knock-in efficiency of a short epitope tag sequence using a similar strategy and found that TIDE-based TIDER outperformed the other tools for this purpose. Taken together, these findings suggest that appropriate tools should be used depending on the type of genome editing.

## 2. Materials and Methods

### 2.1. Preparation of CRISPR–Cas9 and Cas12a RNP Complexes

The CRISPR–Cas9 RNP complexes were prepared as previously described [21]. The crRNAs (Alt-R CRISPR–Cas9 crRNA; listed in Supplementary Table S1), tracrRNAs (Alt-R CRISPR–Cas9 tracrRNA-ATTO 550), and the Cas9 protein (Alt-R S.p. Cas9 Nuclease V3) were purchased from IDT (Coralville, IA, USA). In brief, 3 μM gRNA composed of crRNA and tracrRNA was combined with an equal volume of 3 μM Cas9 to assemble a 1.5 μM RNP complex.

CRISPR–Cas12a RNP complexes were prepared using crRNAs (Alt-R CRISPR–Cas12a crRNA; listed in Supplementary Table S1) and the Cas12a protein (Alt-R A.s. Cas12a Nuclease Ultra) purchased from IDT. The 3 μM crRNA solution was combined with an equal volume of 3 μM Cas12a diluted in Cas9 working buffer (20 mM HEPES pH 7.5, 150 mM KCl) and incubated at 37 °C for 10 min to assemble the RNP complex. Just before microinjection, the RNP complex was mixed with mRNA encoding the Venus fluorescent protein to monitor the success of the microinjection, resulting in an injection solution consisting of 1 μM CRISPR–Cas12a RNP complex and 40 ng/μL Venus mRNA. Venus mRNA was prepared as previously described [22].

### 2.2. Microinjection and Genomic DNA Preparation

At the 1-cell stage, 1 nL of Cas9 or 1.5 nL of the Cas12a RNP complex (1.5 fmol RNP complex) was microinjected into the yolk of Tüpfel long-fin (TL) zebrafish embryos. Embryos at 1 day post-fertilization were lysed in 20 μL/embryo of genomic DNA extraction buffer (10 mM Tris-HCl pH 8, 0.1 mM EDTA, 0.2% [*v*/*v*] Triton-X, 200 mM NaCl, and 0.2 mg/mL proteinase K) and heated at 55 °C for 2–3 h to dissolve the embryos. After heating at 95 °C for 10 min to inactivate protease K, the crude genomic DNA solution was directly subjected to polymerase chain reaction (PCR).

### 2.3. Cloning of Indels Induced by Cas9 and Cas12a

Genomic DNA fragments encompassing the target sites of the crRNAs (Cas9 otx2b_AA/Cas12a otx2b_02, Cas9 pax2a_AB, Cas9 pou2_AG, Cas12a sox2_01, Cas9 sox3_AA, Cas9 sox11a_AB, Cas12a sox11b_01, and Cas12a sox19b_01) were amplified using KOD One PCR Master Mix (Toyobo, Osaka, Japan) and the primers listed in Supplementary Table S2. These fragments were cloned into the pUC19 vector using the restriction sites included in the primers. Sanger sequencing was performed to identify the indel sequences.

### 2.4. Construction of Knock-In Donor Plasmids

To construct the knock-in donor plasmids, the DNA fragments of the left and right homology arms were amplified using specific primers for the *pax2a*, *pou2*, *sox11a*, and *sox3* genes (the primer sequences are listed in Supplementary Table S2) and the pUC clones containing the wild-type sequence of the respective genes serving as templates. For *pax2a*, *pou2*, and *sox3*, the reverse primers used for amplifying the 5′ arm included a FLAG-tag encoding sequence for C-terminal tagging. For *sox11a*, the forward primer used for amplifying the 3′ arm included a FLAG-tag encoding sequence for N-terminal tagging. The 5′ and 3′ arm fragments were simultaneously cloned into a pUC19 vector to obtain the donor plasmid (e.g., pUC19-[pax2a-5′ arm]-FLAG-[pax2a-3′ arm]).

### 2.5. Preparation of Indel Mixtures for the Artificial Sanger Sequencing Template

To prepare artificial template samples for Sanger sequencing, we initially amplified CRISPR–Cas target sequences for the wild-type, various indels, and the knock-in templates using KOD One PCR Master Mix, the pUC universal primers listed in Supplementary Table S2, and corresponding pUC19 clones as templates. The PCR products were purified using NucleoSpin Gel and PCR Clean-up columns (MACHEREY-NAGEL, Düren, Germany). The purified PCR products were subsequently combined in various ratios to mimic real genome-edited samples. These artificial samples were then subjected to Sanger sequencing by a commercial vendor (Eurofin Genomics, Tokyo, Japan) using the primers listed in Supplementary Table S2.

### 2.6. Estimation of Indel Frequencies by Computational Tools

The Sanger sequencing raw data were processed using KB Basecaller (Thermo Fisher Scientific, Waltham, MA, USA) to determine the base calls. The resulting trace data, in the ab1 file format, were subsequently analyzed using TIDE (version 3.3.0; indel size range, 15 and 35 for indels induced by Cas9 and Cas12a, respectively; https://tide.nki.nl (accessed on 1 November 2023)), ICE (v3; https://ice.synthego.com (accessed on 1 November 2023)), DECODR (https://decodr.org (accessed on 1 November 2023)), and SeqScreener (https://apps.thermofisher.com/apps/gea-web/#/setup (accessed on 1 November 2023)) to estimate the indel frequencies. It is important to note that the Sanger sequencing trace data base-called using PeakTrace should not be used as it tends to treat low-level indels as sequencing noise, resulting in an underestimation of the indel frequency.

### 2.7. Zebrafish Husbandry

Zebrafish (*Danio rerio*) were bred and maintained under standard laboratory conditions following a 14-h light/10-h dark cycle. All zebrafish experiments were conducted in compliance with the Fundamental Guidelines for Proper Conduct of Animal Experiments and Related Activities in Academic Research Institutions under the jurisdiction of the Ministry of Education, Culture, Sports, Science and Technology of Japan, using protocols approved by the Animal Experiments Committee of Kochi University of Technology.

## 3. Results and Discussion

### 3.1. Design of a Quantitative Assessment of Computational Tools for Indel Analysis

**Figure 1:**
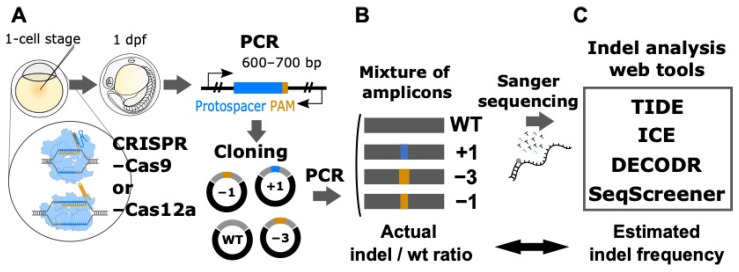
Experimental design for the quantitative assessment of Sanger sequencing-based computational tools for indel analysis. (**A**) Cloning of genomic DNA fragments containing indels induced by CRISPR–Cas9 and CRISPR–Cas12a. Various indels that were induced by CRISPR–Cas9 and CRISPR–Cas12a in several zebrafish genes were PCR-amplified and subsequently cloned into the pUC19 plasmid. (**B**) Preparation of artificial genome-edited samples. The insert DNAs containing predefined indels or the wild-type (wt) sequence were PCR-amplified and purified. These purified PCR products were mixed at varying indel/wt ratios and used as artificial genome-edited samples for Sanger sequencing. (**C**) Computational tool analysis. The resulting Sanger sequencing trace data that were base-called by the KB Basecaller were used as input for the four computational tools TIDE, ICE, DECODR, and SeqScreener, after which the outputted indel frequencies were compared to the actual values.

We aimed to quantitatively assess the performance of computational tools for indel analysis by using predetermined indels as artificial genome-edited samples (Figure 1). To achieve this, we first collected various indels that were induced by CRISPR–Cas systems in several zebrafish genes. Since the type of indels may affect the performance of computational tools, we utilized CRISPR–Cas9 and CRISPR–Cas12a as these two systems are known to generate different types of indels. CRISPR–Cas9 primarily induces short indels, whereas CRISPR–Cas12a tends to produce larger indels, typically with deletions exceeding 10 bp in length [23,24]. To prepare artificial template samples for Sanger sequencing, we mixed wild-type (wt) sequences with one to six indels in various ratios to mimic real samples. The resulting trace data, which were base-called using KB Basecaller, served as input for the four computational tools TIDE, ICE, DECODR, and SeqScreener. Subsequently, the outputted indel frequencies were compared to the actual values. The performance was evaluated using the root mean square error (RMSE) between the estimated and actual values of the indel frequencies for each gRNA and indel mixture type.

### 3.2. Cloning Genomic DNA Fragments Containing Indels Induced by Genome Editing

**Figure 2:**
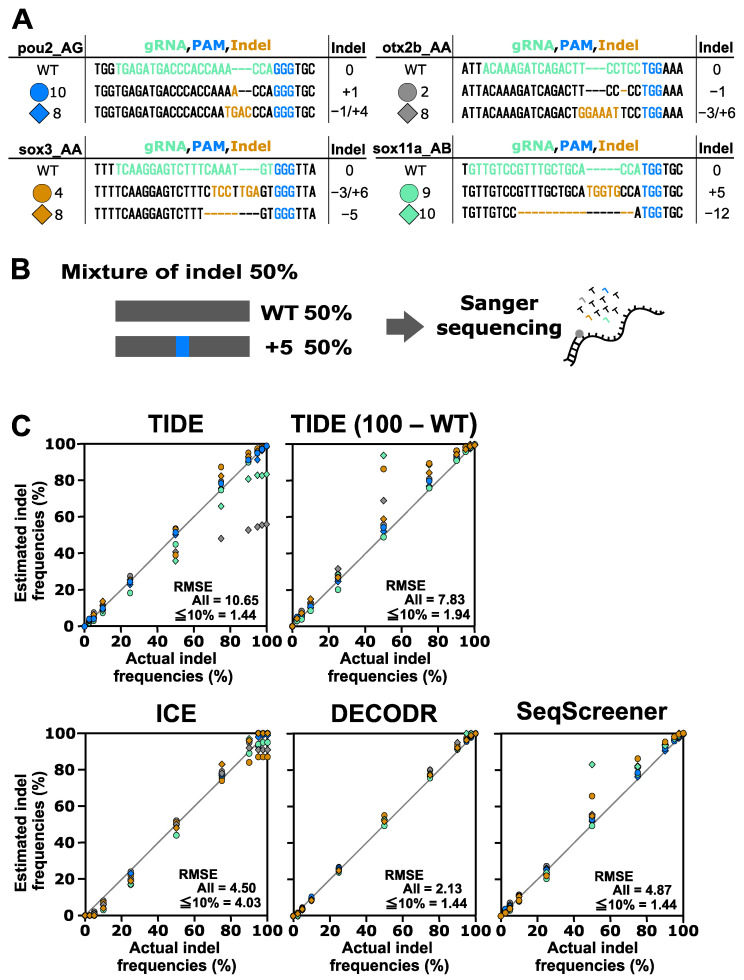
Performance of computational tools for simple mixtures containing an indel sequence induced using Cas9. (**A**) Indel sequences used in this analysis that were induced using Cas9 at the *otx2b*, *pou2*, *sox3*, and *sox11a* genes complexed with corresponding gRNAs, otx2b_AA, pou2_AG, sox3_AA, and sox11a_AB, respectively. The gRNA spacer sequences and the protospacer adjacent motif (PAM) sequence NGG are shown in green and blue, respectively. Inserted nucleotides are shown in orange, and deleted nucleotides are represented by dashed marks in orange. (**B**) An example of an artificial genome-edited sample representing 50% indels. (**C**) Scatter plots comparing indel frequencies (in percent) between the actual values and values estimated by TIDE, ICE, DECODR, and SeqScreener. In the TIDE (100 − WT) plot, values obtained by subtracting the wt percentage from 100% were used. The root mean square error (RMSE) values are shown for all data points as well as specifically for the subset of data points within the 2.5–10% indel range in each plot. The RMSE values for each indel type and all the datasets used for the plots are provided in Supplementary Table S3.

**Figure 3:**
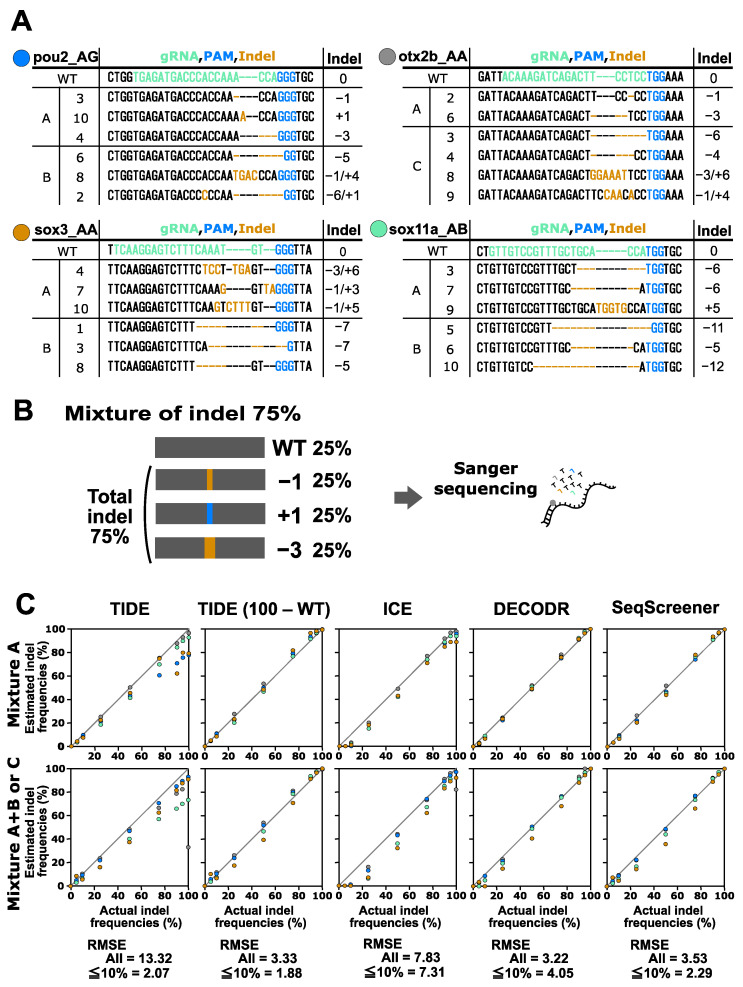
Performance of computational tools for complex mixtures containing two to six indel sequences induced by Cas9. (**A**) Indel sequences used in this analysis were induced using Cas9 as described in Figure 2. Mixtures of group A contained two to three indels. Mixtures of group B that contained three indels were used in combination with mixtures of group A. Mixture C for otx2b_AA was used separately. (**B**) An example of an artificial genome-edited sample representing 75% indels. (**C**) Scatter plots comparing indel frequencies (in percent) between the actual values and values estimated by TIDE, ICE, DECODR, and SeqScreener. In the TIDE (100 − WT) plot, values obtained by subtracting the wt percentage from 100% were used. The upper and lower panels show plots for mixture A and mixture A+B or C, respectively. The RMSE values are shown for all data points as well as specifically for the subset of data points within the 5–10% indel range in each tool. The RMSE values for each mixture type and all the datasets used for the plots are provided in Supplementary Table S3.

**Figure 4:**
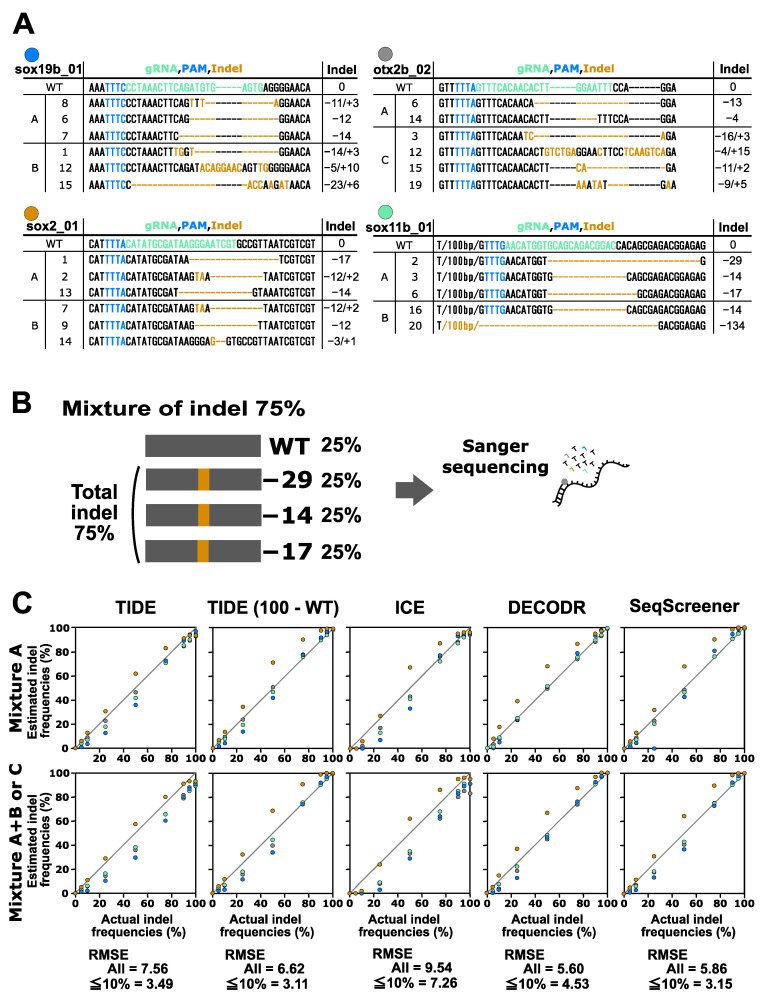
Performance of computational tools for complex mixtures containing two to six indel sequences induced by Cas12a. (**A**) Indel sequences that were induced by Cas12a at the *otx2b*, *sox2*, *sox11b*, and *sox19b* genes complexed with the corresponding gRNAs otx2b_02, sox2_01, sox11b_01, and sox19b_01, respectively. The gRNA spacer sequences and the PAM sequence TTTV are shown in green and blue, respectively. Inserted nucleotides are shown in orange, and deleted nucleotides are represented by dashed marks in orange. Mixtures A, B, and C are as described in Figure 3. (**B**) An example of an artificial genome-edited sample representing 75% indels. (**C**) Scatter plots comparing indel frequencies (in percent) between the actual values and values estimated by TIDE, ICE, DECODR, and SeqScreener. In the TIDE (100 − WT) plot, values obtained by subtracting the wt percentage from 100% were used. The upper and lower panels show plots for mixture A and mixture A+B or C, respectively. The RMSE values are shown for all data points as well as specifically for the subset of data points within the 5–10% indel range in each tool. The RMSE values for each mixture type and all the datasets used for the plots are provided in Supplementary Table S3.

We first cloned various indels that were induced by CRISPR–Cas9 or CRISPR–Cas12a in several zebrafish genes. To achieve this, we injected CRISPR–Cas9 RNP complexes containing one of the gRNAs targeting *pax2a*, *otx2b*, *pou2*, *sox3*, or *sox11a* (Supplementary Table S1) into zebrafish embryos at the one-cell stage. For CRISPR–Cas12a, we used gRNAs targeting *otx2b*, *sox2*, *sox11b*, or *sox19b* (Supplementary Table S1). Genomic DNA was extracted from the injected embryos at 1 day post-fertilization, and the surrounding sequences were amplified through PCR (Figure 1). The PCR amplicons were subsequently cloned into the pUC19 vector and subjected to Sanger sequencing. As expected, the indels induced by CRISPR–Cas9 were mostly short deletions and/or insertions, whereas the majority of the indels induced by CRISPR–Cas12a contained larger deletions and/or insertions, where typical indels contained more than 10 base deletions, with the largest one being a 134-base deletion (Figure 2, Figure 3 and Figure 4). The PCR-amplified and purified DNAs containing these various indels and the wild-type (wt) sequence were mixed at varying indel/wt ratios and used as artificial genome-edited samples for Sanger sequencing.

### 3.3. Performance Evaluation of Computational Tools for Indel Estimation

To evaluate the performance of indel analysis tools for indels induced by CRISPR–Cas9, we used indels obtained from four Cas9 gRNAs targeting the *otx2b*, *pou2*, *sox3*, and *sox11a* genes (Figure 2). We first tested the performance of these tools by mixing one of the indel sequences with their wild-type (wt) sequences at various ratios, representing the simplest scenario. The tested indel percentages were 0%, 2.5%, 5%, 10%, 25%, 50%, 75%, 90%, 95%, 97.5%, and 100%. After Sanger sequencing of these mixtures, the resulting trace data were inputted into the indel analysis tools using the wt trace data as a reference. The output data, representing the estimated indel frequency values, were plotted against the actual indel frequency values (Figure 2). These plots revealed that the four indel analysis tools generally performed well, with some exceptions, but they provided different indel frequency values, especially for samples with low or high indel frequencies. This observation is consistent with recent findings suggesting significant variability in indel frequency values among TIDE, ICE, and DECODR [20]. The RMSE values for all samples indicated that DECODR estimated the indel frequencies most accurately, followed by ICE, SeqScreener, and TIDE. TIDE and SeqScreener yielded erroneous values for larger or compound indels, as exemplified by the indels *otx2b* #8, *sox3* #4, and *sox11a* #10. In these cases, TIDE reported indel frequencies that were much lower than expected, likely due to a unique calculation process that excluded mis-decomposed indels with low *p*-values from the total indel frequency values. In fact, when the total indel frequencies were recalculated by assigning the remaining part of the wt percentages, where TIDE (100 − WT) values were obtained by subtracting the wt percentages from 100%, they aligned better with the actual values, indicating that the wt percentage values were more reliable in TIDE outputs. Interestingly, these recalculated values were similar to those obtained by SeqScreener, suggesting that these two tools employ a similar algorithm. In contrast, TIDE and SeqScreener produced more accurate values for samples with low indel frequencies (2.5% and 5%) than ICE or DECODR, where ICE and DECODR often overlooked low-level indels (Figure 2, Supplementary Table S3). This also suggests that the detection limit of indels may be a few percent when using TIDE and SeqScreener.

Next, we conducted a performance test on the indel analysis tools using a more complex scenario (mixture A) where two or three indel sequences were mixed with the corresponding wt sequence at various ratios (Figure 3). The indel sequence percentages used in this test were 0%, 5%, 10%, 25%, 50%, 75%, 90%, 95%, and 100%. To achieve these percentages, equal proportions of the respective indels were included in each point (a 75% indel mixture is shown in Figure 3 as an example). After Sanger sequencing and computational analysis, the estimated indel frequency values were plotted against the actual indel frequency values (Figure 3). The plots revealed that the four indel analysis tools generally performed well; however, TIDE reported indel frequencies that were much lower than expected for samples with high indel frequencies.

We then conducted a performance test on the indel analysis tools using a mixture of four to six indel sequences with their corresponding wt sequences at various ratios, which represented the most complicated scenarios (mixture A+B and mixture C) (Figure 3). The indel sequence percentages used in these complex samples were the same as those used in the mixture A samples. It was observed that all tools tended to underestimate indel frequencies, particularly for the *sox3* A+B and *sox11a* A+B samples, indicating that these tools struggled with accurately identifying larger deletions. The RMSE values for all of the samples indicated that DECODR once again exhibited the best performance, followed by SeqScreener, ICE, and TIDE. ICE often overlooked low-level indels when the expected indel percentages were 5% and 10%.

Finally, we performed a performance test on the indel analysis tools for indels induced by CRISPR–Cas12a. In this test, the indels obtained from the four Cas12a gRNAs for the *otx2b*, *sox2*, *sox11b*, and *sox19b* genes were used (Figure 4). These indels were mixed with the corresponding wt sequences in various ratios. Two types of mixtures were prepared for this experiment: mixtures containing two or three indel sequences (mixture A) and mixtures containing four to six indel sequences (mixture A+B and mixture C). The indel sequence percentages used were the same as those used for the CRISPR–Cas9 multi-indel samples. For the CRISPR–Cas12a samples containing larger indels, these tools tended to provide more divergent values compared to the CRISPR–Cas9 samples. The RMSE values for all samples indicated that DECODR performed the best for the Cas12a samples, followed by SeqScreener, TIDE, and ICE. However, the differences among the four tools were smaller than those observed in the CRISPR–Cas9 experiments.

Overall, our data indicated that DECODR consistently provided the most accurate indel frequency values for most of the indel samples among the four indel analysis tools. This observation positions DECODR as the preferred choice for Sanger sequencing-based indel analysis. Furthermore, DECODR has no limitations on the inference window sizes for deletions or insertions, whereas TIDE and ICE have limited window sizes of −50/+50 and −30/+14, respectively (window size information is unavailable for SeqScreener), suggesting an additional advantage of DECODR. These findings are consistent with a recent report indicating that DECODR’s estimation of in vivo generated indels is more strongly correlated with high-throughput sequencing analysis than TIDE and ICE [20]. The lower performance of TIDE in the high indel frequency range may be attributable to its calculation procedure, as described above, in which mis-decomposed indels with low *p*-values are excluded from the total indel frequencies, despite the wild-type percentage values closely approximating the actual values. ICE tended to underestimate indel frequencies in the low indel frequency range possibly due to the treatment of low signal peaks as sequencing noise. It is worth mentioning that in the lower indel frequency range, TIDE and SeqScreener appear to provide more accurate indel frequency values than DECODR, as evidenced by their lower RMSE values when the calculation was restricted to samples with 2.5–10% indels (Figure 2, Figure 3 and Figure 4).

### 3.4. Performance Evaluation of Computational Tools for Deconvoluting Indel Sizes and Compositions

The indel analysis tools provide information about the sizes, compositions, and frequencies of indels as well as the total indel frequencies. To assess the effectiveness of these tools in determining indel sizes in complex mixtures containing Cas9 or Cas12a-induced indels, we examined their accuracy in identifying the constituents of artificial samples, specifically mixtures A+B or C, each comprising a total of 75% indels (refer to Figure 3 and Figure 4). For the sake of simplicity in data analysis, we only considered the net size changes resulting from simultaneous insertions and deletions.

As outlined in Table 1, the four computational tools effectively decomposed all constituents of the mixtures, except for the 134-bp deletion induced by Cas12a (sox11b_01, clone #20), indicating their high proficiency in deconvolution even when handling complex indel sequences. We observed discrepancies in the frequencies of individual indels, which appear to stem from inaccuracies in estimating the quantities of individual indels and/or the extent of mis-decomposed sequences. This discrepancy was particularly noticeable in the Cas9 *pou2* A+B and Cas12a *otx2b* C samples, where TIDE and ICE reported significantly lower percentages for specific indels (Cas9 *pou2* clone #4, Cas12a *otx2b* clone #12). The RMSE values for all samples indicated that DECODR exhibited superior performance in deciphering indels induced by both Cas9 and Cas12a, while TIDE, ICE, and SeqScreener showed similar performances.

**Figure 5:**
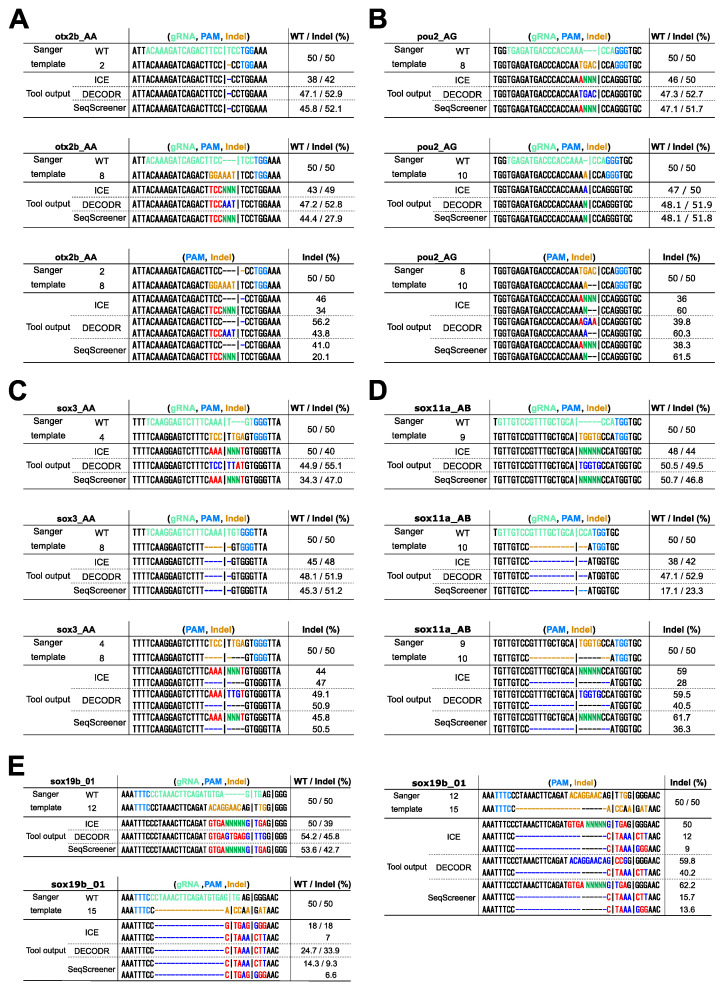
Comparison of the deconvolution capability of indel sequences in clonal cell analysis. Sanger sequencing was performed on 50/50 mixtures of wt/indel and indel/indel sequences, mimicking monoallelic and biallelic editing events, respectively. The indels used were as follows: (**A**) *otx2b* Cas9 indels #2 and #8, (**B**) *pou2* Cas9 indels #8 and #10, (**C**) *sox3* Cas9 indels #4 and #8, (**D**) *sox11a* Cas9 indels #9 and #10, (**E**) *sox19b* Cas12a indels #12 and #15. The resulting trace data were analyzed using ICE, DECODR, and SeqScreener, with the clonal sample type option selected in DECODR and SeqScreener. Each panel displays the alignment of actual sequences with the outputted indel sequences and their frequencies (minor alleles with low frequencies are not shown). In the Sanger template sequences, the gRNA spacer and the PAM sequences are shown in light green and light blue, respectively. Inserted nucleotides are shown in orange, and deleted nucleotides are represented by dashed marks in orange. In the tool output sequences, correctly and incorrectly identified inserted/deleted nucleotides are shown in bright blue and red, respectively. The inserted unknown nucleotides (Ns) are shown in bright green. In the wt/indel outputs, only the estimated indel sequences are shown, as the wt sequences were all correct.

When applying these computational tools to clonal cells derived from genome editing, one might wish to identify exact indel sequences. Among the four tools, only DECODR is designed to comprehensively deconvolute both inserted and deleted sequences. ICE and SeqScreener are designed to deconvolute deleted sequences, but they output extra nucleotide sequences as unknown nucleotides (Ns). TIDE, on the other hand, does not provide indel sequence information except for a single base-pair insertion, and hence, it was excluded from this comparison. To simulate clonal cell analysis, we used 50/50 mixtures of wt/indel and indel/indel sequences, representing monoallelic and biallelic editing events, respectively, and evaluated the effectiveness of these tools in determining indel sequences (Figure 5). When using 50/50 mixtures of wt/indel, all three tools successfully estimated simple deletions, as expected (indels *otx2b* #2, *sox3* #8, and *sox11a* #10). Simple insertions were also well decomposed by these tools, with DECODR accurately predicting inserted sequences (indels *pou2* #10 and *sox11a* #9). However, for composite indels resulting from combined insertion and deletion events, even DECODR outputted erroneous sequences (all the other indels). In ICE and SeqScreener, the inserted sequences were incorrectly presented as the wt sequence along with additional inserted nucleotides represented as unknown nucleotides (Ns). When using 50/50 mixtures of indel/indel, the accuracy of sequence prediction by DECODR decreased in the composite indel sample *pou2* #8/#10 but rather increased in *sox19b* #12/#15. These data indicate that the current ability of these tools to estimate indel sequences is limited, suggesting that the decomposition algorithm may need improvement to fully deconvolute complex indels.

### 3.5. Performance Evaluation of Computational Tools for Estimating Knock-in Frequency

**Figure 6:**
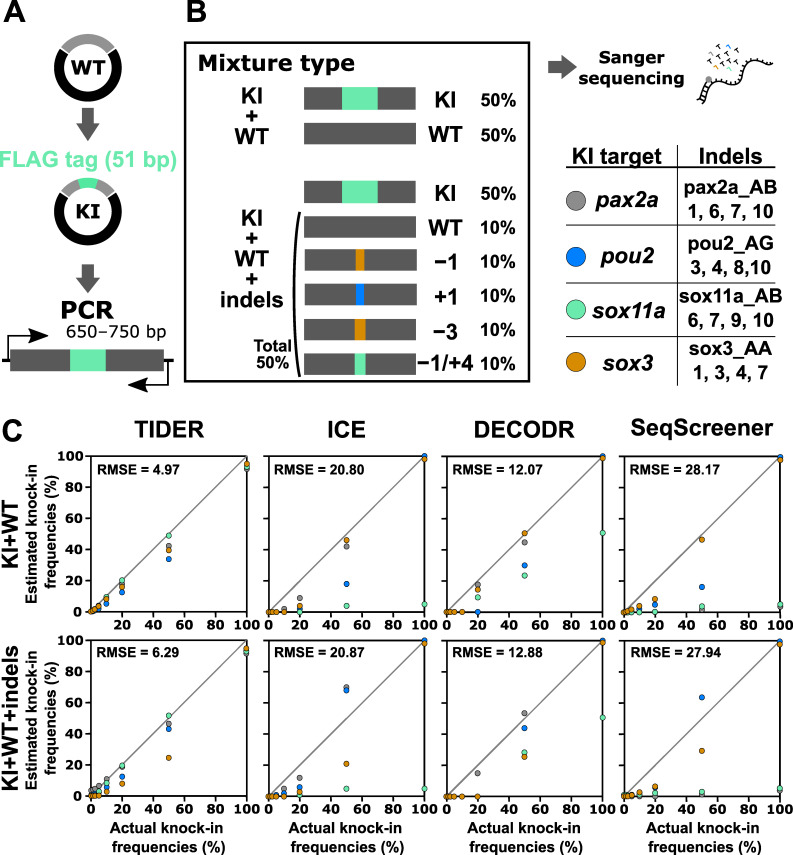
Performance of computational tools for mixtures containing a knock-in sequence with or without indel sequences induced by Cas9. (**A**) A schematic illustration of the knock-in sequences for the *pax2a*, *pou2*, *sox11a*, and *sox3* genes, which contain the FLAG-tag sequence at the beginning or end of the coding sequences. (**B**) Examples of artificial genome-edited samples representing 50% knock-in with or without indels. The indel sequences used in this analysis are shown as clone numbers, the indel sequences of which are shown in Supplementary Table S4. (**C**) Scatter plots comparing knock-in frequencies (in percent) between the actual values and values estimated by TIDER, ICE, DECODR, and SeqScreener. The upper and lower panels show plots for mixtures without indels and mixtures with indels, respectively. The RMSE values are shown in each plot. The RMSE values for each indel type and all the datasets used for the plots are provided in Supplementary Table S3.

The indel analysis tools evaluated above were also designed to estimate knock-in frequencies. A TIDE-based tool modified for knock-in analysis is called TIDER [25]. We therefore tested the performance of the computational tools for knock-in analysis using a similar strategy, where artificial samples that mimic genome editing outcomes of the knock-in procedure were prepared by mixing knock-in sequences with wt and indel sequences (Figure 6). In this experiment, a 51 bp sequence containing the FLAG-tag-encoding sequence was inserted at the beginning or end of the coding sequences of four zebrafish genes: *pax2a*, *pou2*, *sox11a*, and *sox3*. First, these knock-in sequences were mixed with only the wt sequence at various ratios as a simple case, although such mixtures (i.e., mixtures without indels) would not be produced in real knock-in experiments. The knock-in sequence percentages used were 0%, 1.25%, 2.5%, 5%, 10%, 20%, 50%, and 100%. We focused on a low range because knock-in frequencies are generally low. After Sanger sequencing of these mixtures, the resulting trace data were inputted into computational tools with Sanger trace data (for TIDER) or simple nucleotide sequences (for the other three) of the knock-in sequences as the second reference. The output data of the estimated knock-in frequency values were plotted against the actual knock-in frequency values (Figure 6). For these artificial knock-in samples, only TIDER was able to estimate reasonable values close to the expected values. Next, the knock-in sequences were mixed with the wt and Cas9-induced indel sequences in varying ratios to mimic a realistic sample (Figure 6). Again, only TIDER provided estimated indel values that were close to the expected values, although it generated more divergent values than those obtained with the indel-omitted mixtures, especially when the samples contained less than 10% knock-in sequences. The superior performance of TIDER for knock-in analysis was confirmed by its lower RMSE values. This variable performance is likely caused by differences in the tool workflow. Only TIDER uses additional Sanger trace data from the knock-in sequence as the second reference, whereas the other three tools only use nucleotide sequences in the text format. Taken together, these findings indicate that only TIDER can be reliably used for knock-in frequency analysis when the knock-in insertion length is more than several dozen base pairs, although detection in a low knock-in range may not be sufficiently accurate.

## 4. Conclusions

In this study, we conducted a comprehensive comparison of four web tools: TIDE, ICE, DECODR, and SeqScreener. We used artificial sequencing templates with predetermined indels to assess their performance in indel frequency estimation and indel sequence deconvolution. Our systematic investigation revealed that these four computational tools consistently provided indel frequency estimates within an acceptable range for the majority of the tested indel mixtures. Notably, DECODR exhibited superior prediction accuracy across most samples, particularly when the samples contained a high percentage of indels. Consequently, DECODR is currently the preferred choice for Sanger sequencing-based indel frequency analysis, especially when the expected indels exceed the detection size limits of TIDE (−50/+50) and ICE (−30/+14). However, in scenarios where a very low indel frequency range (<10% total indels) is anticipated, TIDE and SeqScreener may offer more accurate indel frequency values than DECODR. Moreover, DECODR is particularly valuable when indel sequence information is necessary for clonal cell analysis, as it has the unique capability to predict inserted sequences, despite some limitations in accuracy for composite indels. In the context of knock-in frequency analysis, TIDER can be considered the primary choice, with a note of caution when interpreting low estimated values for knock-in percentages. Based on these findings, we recommend the thoughtful selection of a tool according to the specific requirements of the genome editing approach.

## Figures and Tables

**Table 1:** Comparison of the deconvolution accuracy of indel sizes and their frequencies.

Cas	gRNA	Indel Mixture	Clone #	Deletion	Insertion	Net Change	Actual Indel Frequency (%)	Estimated Indel Frequency (%)			
								TIDE	ICE	DECODR	Seq Screener
Cas9	otx2b _AA	C	wt				25.0	18.9	22.0	23.7	23.5
			8	−3	6	3	37.5	23.4	36.0	38.0	28.6
			9	−1	4	3					
			4	−4		−4	18.8	17.3	19.0	19.4	21.5
			3	−6		−6	18.8	16.0	18.0	18.8	18.6
						Others	0.0	9.9	0.0	0.0	0.1
	pou2 _AG	A+B	wt				25.0	20.2	23.0	23.2	23.0
			3	−1		−1	12.5	8.5	9.0	9.4	9.4
			10		1	1	12.5	8.1	9.0	10.7	10.6
			4	−3		−3	12.5	4.9	11.0	11.6	7.5
			8	−1	4	3	12.5	9.5	9.0	11.5	11.0
			6	−5		−5	25.0	33.9	36.0	33.4	35.2
			2	−6	1	−5					
						Others	0.0	4.5	0.0	0.0	3.4
	sox3 _AA	A+B	wt				25.0	29.3	32.0	31.8	33.9
			7	−1	3	2	12.5	6.4	9.0	10.4	9.1
			4	−3	6	3	12.5	16.8	18.0	17.7	16.7
			10	−1	5	4	12.5	14.5	13.0	15.1	13.8
			8	−5		−5	12.5	13.3	11.0	12.0	12.7
			1	−7		−7	25.0	11.5	11.0	13.0	13.7
			3	−7		−7					
						Others	0.0	0.0	0.0	0.0	0.1
	sox11a _AB	A+B	wt				25.0	21.6	25.0	24.4	26.6
			6	−5		−5	12.5	12.1	18.0	17.2	18.4
			9		5	5	12.5	11.4	10.0	14.4	13.5
			3	−6		−6	25.0	13.1	15.0	14.2	14.5
			7	−6		−6					
			5	−11		−11	12.5	5.5	7.0	8.9	8.5
			10	−12		−12	12.5	12.2	15.0	16.5	15.1
						Others	0.0	2.5	2.0	4.3	3.5
							RMSE	6.09	4.79	4.27	4.89
Cas12a	otx2b_02	C	wt				25.0	24.5	26.0	26.6	24.8
			19	−9	5	−4	18.8	17.4	18.0	20.6	19.9
			15	−11	2	−9	18.8	17.8	21.0	21.3	20.7
			12	−4	15	11	18.8	15.6	7.0	15.2	15.2
			3	−16	3	−13	18.8	14.0	15.0	16.4	16.0
						Others	0.0	0.0	1.0	0.0	4.6
	sox2_01	A+B	wt				25.0	9.3	9.0	12.8	10.3
			14	−3	1	−2	12.5	11.5	10.0	12.1	12.1
			2	−12	2	−10	25.0	29.7	34.0	31.8	35.1
			7	−12	2	−10					
			9	−12		−12	12.5	10.9	13.0	12.1	12.3
			13	−14		−14	12.5	14.4	16.0	16.3	16.1
			1	−17		−17	12.5	13.7	13.0	14.9	14.2
						Others	0.0	0.0	0.0	0.0	0.5
	sox11b_01	A+B	wt				25.0	24.5	23.0	25.4	25.0
			3	−14		−14	30.0	34.2	35.0	35.3	37.7
			16	−14		−14					
			6	−17		−17	15.0	15.9	17.0	19.3	19.2
			2	−29		−29	15.0	15.6	16.0	20.0	17.1
			20	−134		−134	15.0	0.0	0.0	0.0	0.0
						Others	0.0	0.0	0.0	0.0	0.8
	sox19b_01	A+B	wt				25.0	26.0	27.0	24.1	27.0
			12	−5	10	5	12.5	12.5	12.0	13.4	14.7
			8	−11	3	−8	12.5	10.6	9.0	12.3	13.9
			1	−14	3	−11	12.5	12.6	15.0	12.3	12.9
			6	−12		−12	12.5	9.7	12.0	14.0	12.7
			7	−14		−14	12.5	11.5	11.0	14.0	13.1
			15	−23	6	−17	12.5	3.5	5.0	9.9	5.8
						Others	0.0	0.0	0.0	0.0	0.0
							RMSE	4.82	5.53	4.46	5.27

## Data Availability

All of the data including the Sanger sequencing raw data will be shared upon reasonable request to the corresponding author.

## Supplementary Materials

The following supporting information can be downloaded at: https://www.mdpi.com/article/10.3390/cells13030261/s1, Supplementary Table S1: crRNAs and their protospacer target sequences used in this study; Supplementary Table S2: Lists of primers used in this study; Supplementary Table S3: The datasets of computational tool outputs and their analysis used for scatter plots; Supplementary Table S4: Indel sequences induced by CRISPR–Cas9 for artificial knock-in samples. References [21,22] are cited in Supplementary Materials.
